# Src Inhibition Attenuates Neuroinflammation and Protects Dopaminergic Neurons in Parkinson’s Disease Models

**DOI:** 10.3389/fnins.2020.00045

**Published:** 2020-02-18

**Authors:** Hanyu Yang, Lu Wang, Caixia Zang, Yue Wang, Junmei Shang, Zihong Zhang, Hui Liu, Xiuqi Bao, Xiaoliang Wang, Dan Zhang

**Affiliations:** State Key Laboratory of Bioactive Substrate and Function of Natural Medicine, Department of Pharmacology, Institute of Materia Medica, Chinese Academy of Medical Sciences and Peking Union Medical College, Beijing, China

**Keywords:** Src, microglia, neuroinflammation, Parkinson’s disease, neuroprotection

## Abstract

Chronic neuroinflammation is of great importance in the pathogenesis of Parkinson’s disease (PD). During the process of neuroinflammation, overactivated microglia release many proinflammatory factors, which eventually induce neurodegeneration. Inhibition of excessive microglial activation is regarded as a promising strategy for PD treatment. Src is a non-receptor tyrosine kinase that is closely related to tumors. Recently, some reports indicated that Src is a central mediator in multiple signaling pathways including neuroinflammation. The aim of our study was to demonstrate the role of Src in microglial regulation and neuroinflammation. The lipopolysaccharide (LPS)-stimulated BV2 microglia model and the 1-methyl-4-phenyl-1,2,3,6-tetrahydropyridine (MPTP)-induced PD model were applied in this study. The results showed that inhibition of Src could significantly relieve microgliosis and decrease levels of inflammatory factors. Besides, inhibition of Src function reduced the loss of dopaminergic neurons and improved the motor behavior of the MPTP-treated mice. Thus, this study not only verified the critical role of Src tyrosine kinase in neuroinflammation but also further proved that interfering neuroinflammation is beneficial for PD treatment. More importantly, this study shed a light on the hypothesis that Src tyrosine kinase might be a potential therapeutic target for PD and other neuroinflammation-related diseases.

## Introduction

Parkinson’s disease (PD), an age-related progressive neurodegenerative disorder, is characterized by the degeneration of dopaminergic neurons in substantia nigra pars compacta (SNpc) and resultant depletion of dopamine in the striatum (Iliopoulos et al., 2009). The loss of dopaminergic neurons leads to rigidity, tremor, resting, bradykinesia, and other clinical symptoms (Sveinbjornsdottir, 2016). The pathogenesis of PD still remains to be elucidated, although some factors like genetic, environmental, and immunological conditions have been approved to relate to the destruction of dopaminergic neuron in PD. Recently, many research groups have found that chronic neuroinflammation worsens dopaminergic degeneration in PD (Amor et al., 2013; Calvello et al., 2017; Gu et al., 2017). Neuroinflammatory reactions, especially microglial activation, have been found in many PD animal models, including PD transgenic animal models (Miller et al., 2007) and environmental toxin-induced animal models, such as 1-methyl-4-phenyl-1,2,3,6-tetrahydropyridine (MPTP) (Smeyne et al., 2016), rotenone (Sherer et al., 2003), and 6-OHDA (Harms et al., 2011). Similar to animal models, positron emission tomography (PET) imaging of PD patients showed that the level of microglial activation and proinflammatory mediators are markedly elevated in the SNpc during post-mortem examinations (Mogi et al., 1994). These studies supported that neuroinflammation played a vital role in the loss of dopaminergic neurons and the exacerbation of the clinical symptoms. For the past 60 years, most anti-PD drugs like selegiline and bromocriptine mainly aimed to supply dopamine to relieve PD symptoms, but they neither prevented neuronal damage nor delayed the disease progress. Accumulated evidences suggested that inhibition of neuroinflammation could attenuate neurodegenerative progress. Some anti-inflammatory drugs, including minocycline, dexamethasone, and naloxone, showed dramatic protective effects on dopaminergic neurons in PD animal models (He et al., 2001; Kurkowska-Jastrzbska et al., 2004; Iselin-Chaves et al., 2009). Also, several studies showed that there may be a protective effect of non-aspirin non-steroidal anti-inflammatory drug use in PD patients, consistent with the possible neuroinflammatory pathway in PD pathogenesis (Chen et al., 2005; Gao et al., 2011). These studies have supported the idea that inhibition of neuroinflammation through regulating microglial activation might be a therapeutic approach for PD treatment.

In view of the important role of microglia in neuroinflammation and neuronal injury, many researchers tried to discover the potential targets on microglia (Joers et al., 2017; Shin et al., 2018), of which Src has drawn more and more attention. Src is a non-receptor tyrosine kinase and is expressed ubiquitously in all cell types (Stehelin et al., 1976). Besides, Src tyrosine kinase is central mediator in multiple signaling pathways, which modulate physiological activities like maintaining cell homeostasis, intercellular, contacts and cell migration; regulating cell shape; and controlling acute inflammatory response-related signal transduction (Thomas and Brugge, 1997; Sen and Johnson, 2011; Ishizawar and Parsons, 2017). Researchers have found that overexpression or high activated Src frequently occurred in tumor tissues (Sen and Johnson, 2011; Ishizawar and Parsons, 2017). In addition, several scientists also found that Src signaling pathway was involved in the cytokine-regulated crosstalk between tumor cells and inflammatory cells (Iliopoulos et al., 2009; Li et al., 2017). Hence, researchers began to explore whether Src was also associated with other inflammatory diseases. Recently, Socodato et al. (2014) found that Src is sufficient for regulating microgliosis *in vitro* and *in vivo*. Dhawan and Combs (2012), Dhawan et al. (2012) and Manocha et al. (2015) found that family tyrosine kinase inhibitor with anti-inflammatory effects in response to Aβ stimulation of microglia. Therefore, we hypothesized that properly modulating of Src tyrosine kinase activity could regulate microglial activation, which may ameliorate the symptoms of neuroinflammation and be helpful in the treatment of PD and other neuroinflammation-related diseases. In this paper, we used the lipopolysaccharide (LPS)-treated BV2 cell model and MPTP-treated animal model to verify the effect of Src inhibition and related mechanisms on neuroinflammation and neuroprotection *in vitro* and *in vivo*.

## Materials and Methods

### Cell Culture

The BV2 cell line, C6 cell line, and SH-SY5Y cell line were purchased from the Cell Culture Center at the Institute of Basic Medical Sciences, Chinese Academy of Medical Sciences and Peking Union Medical College. Cells were cultured at 37°C in an atmosphere of 5% CO_2_ in Dulbecco’s modified Eagle medium (DMEM; Cat#12100-500, Solarbio, China) supplemented with 10% heat-inactivated fetal bovine serum (FBS; Cat#11011-8611, Sijiqing, China).

Cortices from newborn C57/BL6 mice were dissected into Hanks Balanced Salt Solution (HBSS) on ice and cut into small pieces. Cells were dissociated with 1% DNase I and 2.5% trypsin in HBSS at 37°C. The cell suspension was filtered through a 70-μm cell strainer and seeded into poly-L-lysine-coated (Cat#P1399, Sigma, United States) tissue culture flasks and maintained in DMEM/F12 (Cat#SH30023, HyClone, United States) with 10% FBS (Cat#16000-044, Gibco, United States), 1% glutamax (Cat#35050-061, Gibco, United States), and 1% penicillin–streptomycin for 10–14 days to grow a confluent mixed astrocyte/microglia population. The confluent mixed glia cultures were shaken in an orbital shaker at 37°C with 230 rpm for 2 h. The floating microglia cells were collected, centrifuged (250 × *g* × 10 min), and plated on a poly-L-lysine-coated 24 cell plates (1 × 10^5^ cells per well) and cultured at 37°C in an atmosphere of 5% CO_2_.

### Chemical Reagents

For *in vitro* study, Src family kinase inhibitor PP2 (Cat#HY-13805, MCE, United States) was dissolved in dimethyl sulfoxide (DMSO; Cat#0231, AMRESCO, United States) to obtain a stock solution of 200 mM. And then the stock solution was diluted with cell culture media to an appropriate concentration with the concentration of DMSO lower than 0.1%. For *in vivo* study, PP2 was dissolved in DMSO and then diluted with saline (0.9% NaCl) to the concentration of 2.5 mg/kg with a 1% maximal concentration of DMSO. L-DOPA (Cat#SH2443, Roche, Switzerland) was suspended in 0.5% (w/v) sodium carboxymethyl cellulose (CMC-Na; Cat#C4888, Sigma-Aldrich, United States) to the concentration of 20 mg/kg for *in vivo* oral administration. MPTP (Cat#M0896, Sigma-Aldrich, United States) was dissolved with saline to the concentration of 30 mg/kg before injection.

### Experimental Animals and Treatments

Male C57BL/6 mice (10 weeks old, 25–28 g) obtained from the Animal Center of Chinese Academy of Medical Sciences were kept in a temperature-controlled environment (24°C) under a 12/12-h light/dark cycle, and food and water were available. All protocols and procedures involving animals were approved by the Animal Care and Welfare Committee of Institute of Materia Medica, Chinese Academy of Medical Sciences and Peking Union Medical College.

Mice were randomly divided into four groups (*n* = 15 in each group): control group, MPTP-treated group, L-DOPA group, and PP2 group. Mice were treated with vehicle (1% DMSO and 99% saline, i.p.), L-DOPA (p.o.), or PP2 (i.p.) 30 min before each MPTP hydrochloride injection (i.p.) for seven consecutive days. From day 8 to day 12, mice were only treated with vehicle, PP2, or L-DOPA. At days 7, 10, and 12, we measured the motor function of mice using rotarod tests. Body weights were measured at the beginning and the end of the dosing period (at day 1 and day 13). Researchers blinded to the group assignment performed the behavioral tests. During the experiment, one mouse of PP2 group died at day 8.

### Rotarod Test

The rotarod test is used to measure coordinated motor skills. This test requires animals to maintain balance and keep walking on a rotating cylinder. The mice were positioned on the rotarod, and then the rotarod was set to revolve at 30 rpm for up to 120 s. The latency represented the first time that mice fell off the rod, and that time could be automatically recorded by the rotarod. Researchers who were blinded to the group assignment performed the behavioral tests.

### Cell Viability Assay

The cytotoxicity of PP2 was assessed by MTT assay. BV2 cells were seeded in 96-well plates at a density of 8 × 10^3^ cells per well. After that, cells were treated with or without LPS (1 μg/ml, Cat#L4391, Sigma, United States) or PP2 for 24 h, and then the MTT solution (0.5 mg/ml, Cat#M2128, Sigma, United States) was added to each well. After incubation for 4 h at 37°C in 5% CO_2_, the supernatant was removed, and 150 μl of DMSO was added to solubilize the formazan crystals produced in viable cells. After 10 min, the absorbance of each well was measured at 570 nm using a microplate reader (BioTek, United States).

BV2 cells were seeded in 6-well plates at a density of 2 × 10^6^ cells per well and treated with or without 1 μg/ml of LPS or 20 μM of PP2 for 6 h. Then, conditioned media of the BV2 cells were collected and applied to SH-SY5Y cells that have been seeded in 96-well plates. The changes in SH-SY5Y cell viability were assessed by MTT assay after 24 h. The phase-contrast microscopy was used to examine the morphological change in the SH-SY5Y cells.

### Measurement of Nitric Oxide Concentration

Griess method was used to measure nitric oxide (NO) concentration in cell cultures with microplate. In brief, 100 μl of aliquots from conditioned media and an equal volume of the Griess reagent are mixed and incubated at room temperature for 10 min. Then we used a microplate reader to measure nitrite concentration by measuring the absorbance at 540 nm.

### Nuclear and Cytosolic Protein Extraction

Nuclear and cytosolic protein was separated according to the standard protocol in the manufacturer’s instructions (Cat#DE201-01, TransGen Biotech, China).

### Western Blot Analysis

Cells or tissues were homogenized in radioimmunoprecipitation assay (RIPA) lysate buffer with protease phosphatase inhibitor and protease inhibitor. The total protein concentrations were determined by bicinchoninic acid (BCA) kit (Cat#P1151, Applygen, China) to ensure equal sample loading. Protein contents were separated on SDS–poly-acrylamide gels (10%) and then transferred into a 0.45-μm polyvinylidene fluoride membrane (Cat#IPVH00010, Millipore, United States), which were blocked with 5% skim milk–TBST (20 mM of Tris–HCl, pH 7.5, and 500 mM of NaCl, 0.1% Tween 20) for 1 h. The membranes were probed with the following antibodies: β-actin rabbit antibody (1:10,000, Cat#AC026, ABclonal, China), iNOS rabbit antibody (1:1,000, Cat#ab15323, Abcam, United States), Src rabbit antibody (1:1,000, Cat#2109, Cell Signaling Technology, United States), ionized calcium-binding adaptor molecule-1 (IBA1) rabbit antibody (Cat#A1527, 1:1,000, ABclonal, China), cyclooxygenase-2 (COX2) rabbit antibody (Cat#23672, 1:1,000, Abcam, United States), p-Src-Tyr416 rabbit antibody (1:1,000, Cat#2101, Cell Signaling Technology, United States), tyrosine hydroxylase (TH) rabbit antibody (Cat#2792, 1:1,000, Cell Signaling Technology, United States), IKKα rabbit antibody (Cat#2682, 1:1,000, Cell Signaling Technology, United States), p-IKKα/β-Ser176/180 rabbit antibody (Cat#2697, 1:1,000, Cell Signaling Technology, United States), NF-κB rabbit antibody (Cat#8242, 1:1,000, Cell Signaling Technology, United States), and histone H3 rabbit antibody (Cat#9728, 1:1,000, Cell Signaling Technology, United States) overnight at 4°C; and then these were incubated with horseradish peroxidase (HRP) goat anti-rabbit IgG (H + L) (Cat#AS014, 1:2000, ABclonal, China) for 2 h at room temperature. The blots were visualized by incubating the membranes with ECL Plus reagents (Cat#36208, Yeasen, China), and the images were recorded by LAS-4000 chemiluminescence system (GE Healthcare, United States). The blot densities were assessed by Gel-pro analyzer 4.0.

### Immunocytochemistry Assay

Cells cultured on coverslips were washed thrice by cold phosphate-buffered saline (PBS) for and then fixed in 4% formaldehyde for 15 min. Then we added 0.1% Triton X-100 to permeabilize the cells. After incubation with 3% normal goat serum for 2 h at room temperature, fixed cells were incubated overnight at 4°C with antibodies against IBA1 (1:500, Cat#019-19741, WAKO, Japan) or p-Src-Tyr416 (1:200). Cells were washed with PBS and then incubated for 2 h with goat anti-rabbit IgG H&L (Alexa Fluor^®^ 488) antibody (Cat#ab150077, 1:500, Abcam, United States) or goat anti-rabbit IgG H&L (Alexa Fluor^®^ 647) antibody (Cat#ab150079, 1:500, Abcam, United States) followed by incubation with DAPI for 10 min. Coverslips were imaged by confocal microscopy (Leica, Germany). Quantification was carried out using ImageJ software.

### Immunohistochemistry Assay

Animals were perfused with 0.9% NaCl, and the brains were dissected and post-fixated with 4% paraformaldehyde for 1 day. Then brains were cut coronally into 30-μm thickness. The slides were permeabilized and blocked with 10% donkey serum containing 0.3% Triton X-100 for 0.5 h. After that, they were incubated with primary antibodies against TH (1:50), IBA1 (1:100), and p-Src-Tyr416 (1:100) at 4°C overnight, and then they were incubated with secondary antibodies conjugated with streptavidin-labeled peroxidase followed by DAB coloration. All the slides were scanned using a Pannoramic MIDI Digital Slide Scanner (3DHISTECH, Hungary), and the number of positive cells was calculated by ImageJ software according to the manual. Briefly, the threshold was adjusted to include all of the positive cells, and then particles were analyzed to get the number of positive cells. The SNc region was defined according with the brain anatomy map. The cells with brown stain were defined to be positive. At least three slides were analyzed in each group.

### Quantitative Reverse Transcription–PCR Analysis

Total RNA was isolated using a TransZol Up Plus RNA Kit (Cat#ER501, TransGen, China), and cDNA was synthesized from 1 mg of total RNA using a TransScript One-Step gDNA Removal and cDNA Synthesis SuperMix kit (Cat#AT311, TransGen, China) according to the manufacturer’s instructions. Quantitative reverse transcription (qRT)-PCR was performed using a TransStart Tip Green qPCR SuperMix (Cat#AQ141, TransGen, China) and ABI7900 instrument (Applied Biosystems, United States). The detected expression of mRNA was normalized using β-actin as an internal control. The relative mRNA levels were calculated by the 2^–Δ^ ^Δ^ ^*Ct*^ method. Prime sequences used in the study are shown in Table 1.

**TABLE 1:** Primer sequence.

Gene	Primer sequence (5′–3′)	
	
	Forward	Reverse
TH	AGA CAG CTT CGT GTT TGA GGA GGA	TCT TTC TGC TCG CTC GAC TTT CCA
IBA1	ATC AAC AAG CAA TTC CTC GAT GA	CAG CAT TCG CTT CAA GGA CAT A
IL-6	CTG CAA GAG ACT TCC ATC CAG	AGT GGT ATA GAC AGG TCT GTT GG
TNF-α	TAC TGA ACT TCG GGG TGA TTG GTC C	CAG CCT TGT CCC TTG AAG AGA ACC
β-Actin	GCA CCA CAC CTT CTA CAA	TAC GAC CAG AGG CAT ACA

*TH, tyrosine hydroxylase; IBA1, ionized calcium-binding adaptor molecule-1.*

### ELISA Analysis

The concentration of TNF-α in culture supernatants was measured by ELISA Analysis using mouse TNF-α-precoated ELISA kit (DKW12-2720-096, Dakewe, China) according to the manufacturer’s instructions.

### Statistical Analysis

Data are expressed as the means ± SEM (standard error of mean). Statistically significant differences were performed using the Statistical Package for GraphPad Prism 6.0 software. Statistical analysis was performed using one-way ANOVA followed by Tukey’s multiple-comparison test. *P* < 0.05 was considered to be statistically significant.

## Results

### The Efficiency of Src Inhibitor PP2 Was Confirmed in Lipopolysaccharide-Induced Microglial Activation

**FIGURE 1:**
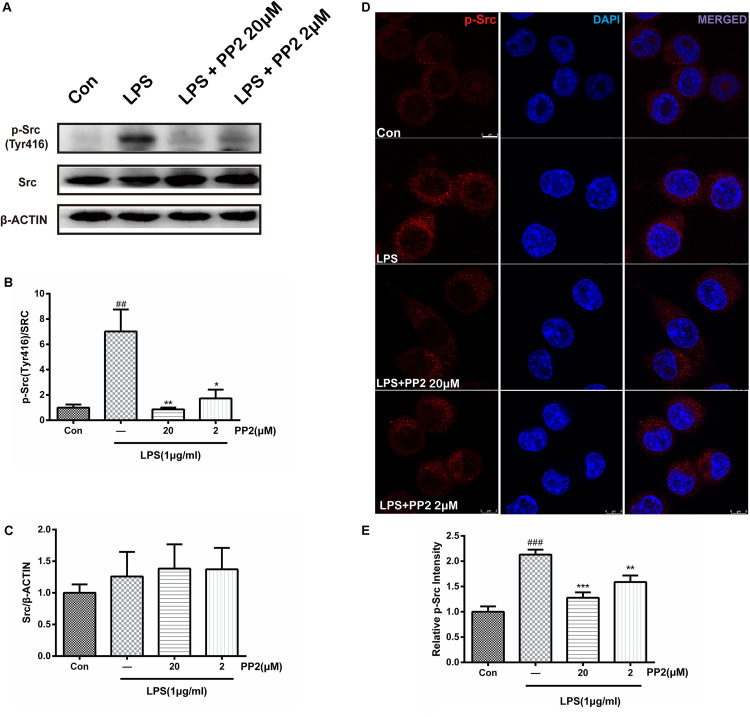
The efficiency of Src inhibitor PP2 was confirmed in lipopolysaccharide (LPS)-induced BV2 activation. Cultured BV2 microglia were treated with two concentrations of PP2 (2 and 20 μM) in the presence of LPS (1 μg/ml) for 24 h. **(A–C)** The protein levels of p-Src and Src were determined by western blot analysis. β-Actin was used as an internal loading control. Each bar represents the mean ± SEM. *n* = 4. ^##^*P* < 0.01 vs. control group,**P < 0.01, and *P < 0.05 vs. LPS group. **(D)** The cells were stained with anti-p-Src antibody (red) and DAPI stain (scale bar: 8 μm). **(E)** The fluorescence intensity of the p-Src staining of was provided in a histogram. Each bar represents the mean ± SEM. *n* = 3. ^###^*P* < 0.001 vs. control group, **P < 0.01 vs. LPS group.

PP2 is a potent inhibitor of Src tyrosine kinases, which could inhibit Src phosphorylation in an ATP-competitive manner. Firstly, we verified the efficiency of PP2 on BV2 microglial cells. Cell viability experiment showed that PP2 at the concentration range of 2–20 μM did not have a toxic effect on cell viability in BV2 microglia with or without 1 μg/ml of LPS, whereas PP2 at 200 μM produced significant cytotoxicity (Supplementary Figure S1). Therefore, we used PP2 at 2 and 20 μM for further study. Then we examined the effect of PP2 on the phosphorylation of Src in LPS-stimulated microglial cells. As shown in Figures 1A–C, western blot analysis revealed that PP2 inhibited LPS-induced Src phosphorylation (Tyr416). The result of confocal imaging analysis was consistent with the result in western blot analysis (Figures 1D,E). The inhibition effect of PP2 was also validated in LPS-induced primary microglial cells (Supplementary Figure S2A). These data confirmed the efficiency of Src inhibitor PP2 in LPS-induced microglial activation.

### Src Inhibition Prevented the Activation of Microglia and the Production of Neuroinflammatory Molecules Subjected to Lipopolysaccharide

**FIGURE 2:**
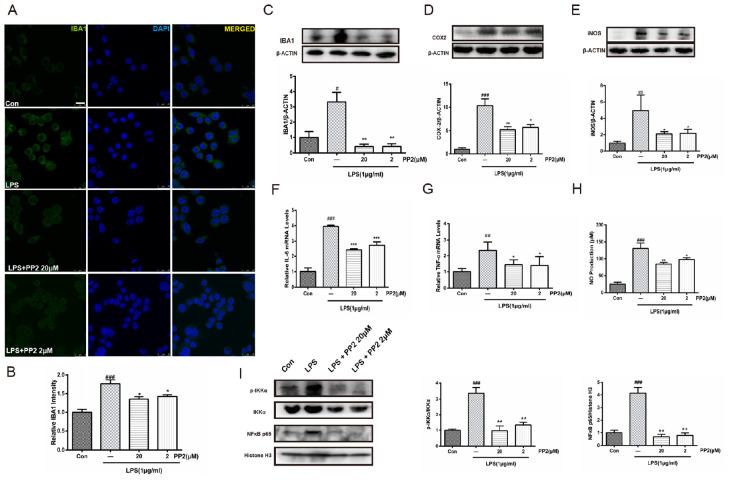
Src inhibition prevented the activation of BV2 microglia and the production of neuroinflammatory molecules subjected to lipopolysaccharide (LPS). **(A)** Cultured BV2 cells were treated with two concentrations of PP2 (2 and 20 μM) in the presence of LPS (1 μg/ml) for 24 h, and then the cells were stained with anti-IBA1 antibody (green) and DAPI stain (scale bar: 8 μm). **(B)** Quantification of the IBA1 staining was provided in a histogram. Each bar represents the mean ± SEM. *n* = 4. ^###^*P* < 0.001 vs. control group, *P < 0.05 vs. LPS group. **(C)** BV2 cells were treated with two concentrations of PP2 (2 and 20 μM) in the presence of LPS (1 μg/ml) for 24 h. The protein level of IBA1 was analyzed by western blot with anti-IBA1 antibody. β-Actin was used as an internal loading control. Each bar represents the mean ± SEM. *n* = 4. ^#^*P* < 0.05 vs. control group,**P < 0.01 vs. LPS group. **(D,E)** The protein level of cyclooxygenase-2 (COX2) and iNOS were examined by western blot. Each bar represents the mean ± SEM. *n* = 4. ^###^*P* < 0.001 and ^##^*P* < 0.01 vs. control group, **P < 0.01 and *P < 0.05 vs. LPS group. **(F,G)** The mRNA levels of IL-6 and TNF-α were analyzed by quantitative reverse transcription (qRT)-PCR. Each bar represents the mean ± SEM. *n* = 4. ^##^*P* < 0.01 and ^###^*P* < 0.001 vs. control group, *P < 0.05 and ***P < 0.001 vs. LPS group. **(H)** The level of NO production was determined using the Griess reaction. Each bar represents the mean ± SEM. *n* = 5. ^###^*P* < 0.001 vs. control group, *P < 0.05 and **P < 0.01 vs. LPS group. **(I)** The protein expression level of p-IKKα, IKKα, NF-κB p65, and histone H3 were measured by western blot. Each bar represents the mean ± SEM. *n* = 4. ^###^*P* < 0.001 vs. control group, **P < 0.01 vs. LPS group.

To determine whether Src inhibition would reduce LPS-triggered microglial activation, IBA1 protein expression was assessed in BV2 microglial cells. Confocal imaging analysis showed that the inhibition of Src significantly depressed LPS-induced IBA1 protein expression (Figures 2A,B), and western blot analysis confirmed this effect (Figure 2C). Given that IBA1 is regarded as a marker of microglia activation, these data indicated that LPS-induced activation of BV2 microglia was prevented by Src inhibition. Then we examined the effects of Src inhibition on the production of inflammatory cytokines. Western blot analysis revealed that LPS incubation with BV2 microglia resulted in an increase in iNOS and COX2 protein expression, and these effects were inhibited in the presence of PP2 (Figures 2D,E). Furthermore, the inhibition of Src decreased the mRNA levels of IL-6 and TNF-α in BV2 microglial cells subjected to LPS (Figures 2F,G). Griess reaction was used to measure NO production. Compared with that in the control group, the release of NO was significantly increased by LPS stimulation, and the Src inhibitor notably reduced NO production (Figure 2H). The nuclear factor NF-κB pathway has long been considered as a prototypical proinflammatory signaling pathway. Thus, we studied the effects of Src inhibition on NF-κB signaling pathway. We found that PP2 could reduce the phosphorylation of IKK-α and nuclear translocation of NF-κB in LPS-treated BV2 cells (Figure 2I), which meant the inhibition of NF-κB pathway. PP2 also decreased TNF-α production in LPS-treated primary microglial cells (Supplementary Figure S2B). To sum up, our data revealed that Src inhibition prevented the secretion of neuroinflammatory molecules in LPS-stimulated microglia.

### Src Inhibition Decreased Neurotoxicity Mediated by Lipopolysaccharide-Stimulated Microglia

**FIGURE 3:**
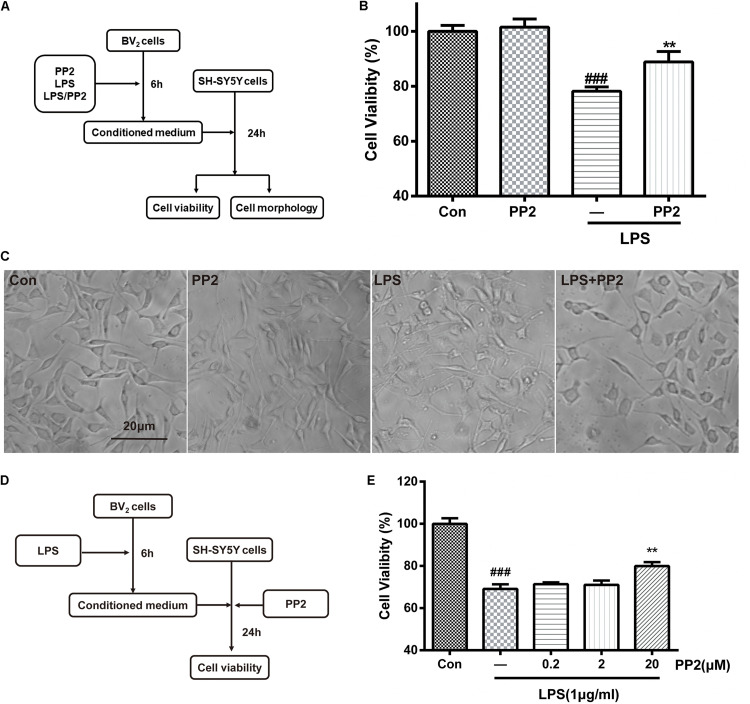
Src inhibition decreased neurotoxicity mediated by lipopolysaccharide (LPS)-stimulated microglia. **(A)** The experimental design. **(B)** The cell viability of SH-SY5Y cells was assessed by MTT assay. Each bar represents the mean ± SEM. *n* = 6. ^##^*P* < 0.01 vs. control group, **P < 0.01 vs. LPS group. **(C)** The morphology of SH-SY5Y cells was observed by the phase-contrast microscope (scale bar: 20 μm). **(D)** The experimental design. **(E)** The cell viability of SH-SY5Y cells was assessed by MTT assay. Each bar represents the mean ± SEM. *n* = 5. ^##^*P* < 0.01 vs. control group, **P < 0.01 vs. LPS group.

It is well-known that activated BV2 cells could release proinflammatory cytokines and other toxic molecules (Yan et al., 2017), which may elicit the death of neurons. To further confirm that Src was involved in microglial activation and the neuroinflammatory molecules production, the neurotoxicity of the conditioned media from microglia was tested. As shown in Figure 3A, BV2 microglial cells were treated with 1 mg/ml of LPS for 6 h with or without PP2 (20 μM) treatment. The conditioned media collected from microglia were subsequently applied to SH-SY5Y culture. Cell viability and cell morphology were analyzed after 24-h treatment. We found that SH-SY5Y cell viability was reduced after incubating with conditioned media from LPS-treated BV2 cells, and the morphological change of SH-SY5Y cells could be observed. The cells tended to be round and detached themselves from the bottom of the well. Also, cell debris appeared. Src inhibition could partially block the effect of neurotoxicity mediated by LPS-stimulated microglia (Figures 3B,C). Because Src is a ubiquitous protein, expressed in almost all cell types including SH-SY5Y, we wonder whether PP2 could directly preserve neurons from death caused by exposure to the conditioned media derived from LPS-treated BV2 cells. As shown in Figure 3D, BV2 microglial cells were treated with 1 mg/ml of LPS for 6 h. The conditioned media collected from microglia were subsequently applied to SH-SY5Y culture with or without PP2 treatment. Cell viability was analyzed after 24-h treatment. We found that Src inhibition in neuron could also block the effect of neurotoxicity mediated by LPS-stimulated microglia (Figure 3E). These data supported that Src is critical for microglia to exert neurotoxic effect.

### The Efficiency of Src Inhibitor PP2 Was Confirmed in MPTP-Treated Mice

**FIGURE 4:**
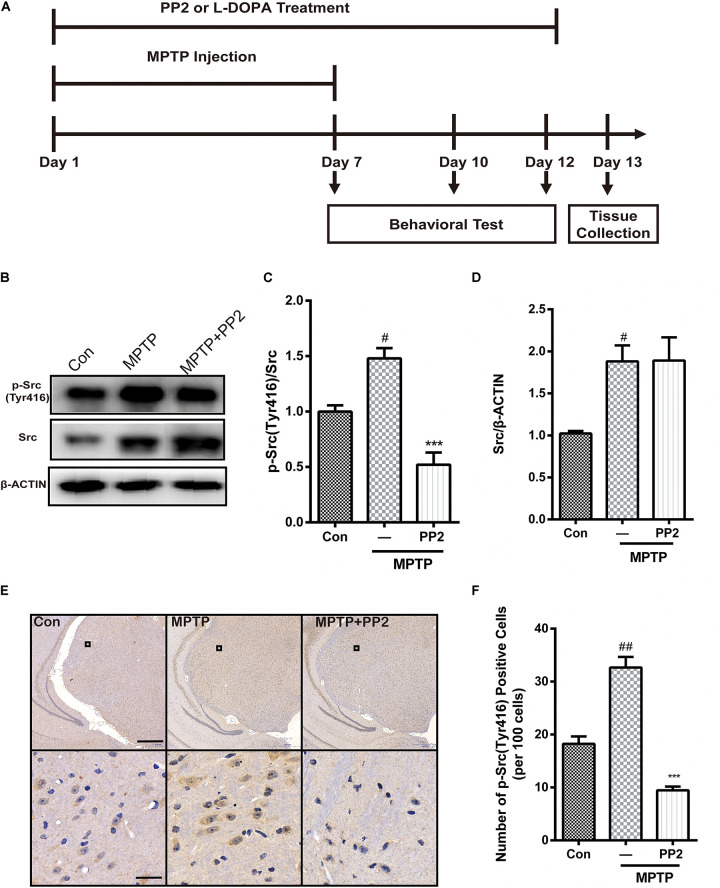
The efficiency of Src inhibitor PP2 was confirmed in 1-methyl-4-phenyl-1,2,3,6-tetrahydropyridine (MPTP)-treated mice. **(A)** The experimental arrangement. **(B–D)** The protein level of p-Src and Src in SNpc of MPTP-treated mice was analyzed by western blot with anti-p-Src and anti-Src antibodies. Data are expressed as means ± SEM. *n* = 4. ^#^*P* < 0.05 vs. control group, ***P < 0.001 vs. MPTP group. **(E)** The brown stain represented p-Src-immunoreactive cells in SNpc (scale bar: top, 600 μm; bottom, 25 μm). **(F)** The number of p-Src-positive cells per 100 cells in SNpc was counted and provided in a histogram. Data are expressed as means ± SEM. *n* = 4. ^#^*P* < 0.05 and ^##^*P* < 0.01 vs. control group, **P < 0.01 and ***P < 0.001 vs. MPTP group.

As shown in Figure 4A, mice were treated with PP2 (2.5 mg/kg) or L-DOPA (20 mg/kg) 30 min before each MPTP hydrochloride (30 mg/kg) injection for seven consecutive days. From day 8 to day 12, mice were only treated with PP2 or L-DOPA. To investigate the efficiency of Src inhibitor PP2 in MPTP-treated mice, the expression of phospho-Src (Tyr416) and Src in SNpc was measured using western blot analysis. We found that the expression of phospho-Src (Tyr416) was significantly increased in the SNpc of MPTP-induced PD model mice. PP2 markedly decreased phospho-Src (Tyr416) expression but had nothing to do with total Src expression (Figures 4B–D). The result of immunohistochemistry analysis was consistent with the result in western blot analysis (Figures 4E,F). These results indicated that PP2 could inhibit Src activation in the *in vivo* study.

### Src Inhibition Reduced Activation of Microglial Cells and Neuroinflammation in MPTP-Treated Mice

**FIGURE 5:**
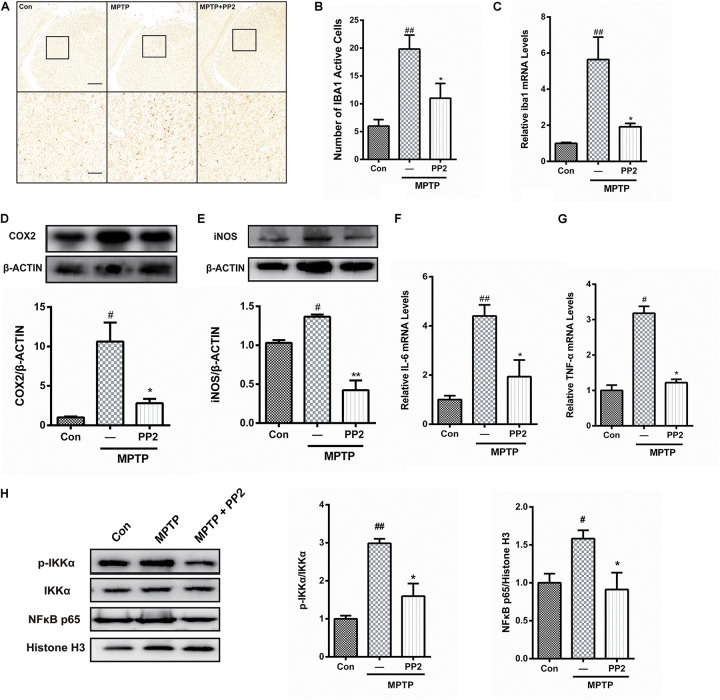
Src inhibition reduced activation of microglial cells and neuroinflammation in 1-methyl-4-phenyl-1,2,3,6-tetrahydropyridine (MPTP)-treated mice. **(A)** The activation of microglia in SNpc showed by IBA1 immunostaining (scale bar: top, 200 μm; bottom, 50 μm). **(B)** The number of IBA1-positive cells per 100 cells was counted and provided in a histogram. Data are expressed as means ± SEM. *n* = 4. ^##^*P* < 0.01 vs. control group *P < 0.05 vs. MPTP group. **(C)** The mRNA expression level of IBA1 in SNpc was determined by quantitative reverse transcription (qRT)-PCR. Data are expressed as means ± SEM. *n* = 4. ^##^*P* < 0.01 vs. control group, *P < 0.05 vs. MPTP group. **(D,E)** The protein expression level of cyclooxygenase-2 (COX2) and iNOS were determined by western blot with anti-COX2 and anti-iNOS antibodies. Data are expressed as means ± SEM. *n* = 4. ^#^*P* < 0.05 vs. control group, *P < 0.05 and **P < 0.01 vs. MPTP group. **(F,G)** The mRNA expression level of IL-6 and TNF-α was determined by qRT-PCR. Each bar represents the mean ± SEM. *n* = 4. ^#^*P* < 0.05 and ^##^*P* < 0.01 vs. control group, *P < 0.05 vs. MPTP group. **(H)** The protein expression level of p-IKKα, IKKα, NF-κB p65, and histone H3 were measured by western blot. Each bar represents the mean ± SEM. *n* = 4. ^#^*P* < 0.05 and ^##^*P* < 0.01 vs. control group, *P < 0.05 vs. MPTP group.

To determine whether Src inhibition was associated with the reduction of microglial activation in MPTP-treated mice, immunohistochemistry analysis of IBA1 expression was carried out in the *in vivo* study. The number of IBA1-positive cells showed a significant increase in the SNpc of MPTP-treated mice, which was markedly decreased by PP2 treatment (Figures 5A,B). Moreover, the result of qRT-PCR was consistent with the result of immunohistochemistry analysis (Figure 5C). As shown in Figures 5D,E, COX−2 and iNOS protein expression levels were apparently reduced when Src was inhibited in MPTP-treated mice. Also, IL-6 and TNF-α mRNA levels were clearly lower in mice treated with Src inhibitor PP2 (Figures 5F,G). Also, we studied the effects of Src inhibition on NF-κB signaling pathway. We found that PP2 could reduce the phosphorylation of IKK-α and nuclear translocation of NF-κB in MPTP-treated mice (Figure 5H). These results indicated that the inhibition of Src reduced the production of neuroinflammatory molecules in MPTP-treated mice, resulting in anti−inflammatory effects.

### Src Inhibition Enhanced the Survival of Dopaminergic Neurons of the MPTP-Treated Mice

**FIGURE 6:**
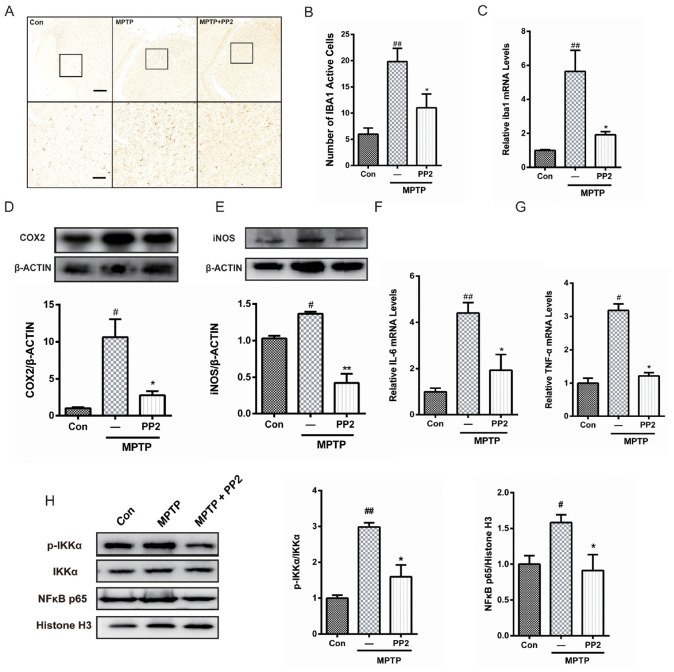
Src inhibition enhanced the survival of dopaminergic neurons of the 1-methyl-4-phenyl-1,2,3,6-tetrahydropyridine (MPTP)-treated mice. **(A)** Representative images showed tyrosine hydroxylase (TH)-immunoreactive neurons in the SNpc (scale bar: top, 500 μm; bottom, 250 μm). **(B)** The number of TH-positive neurons per slide in SNpc was counted for each section and provided in a histogram. Data are expressed as means ± SEM. *n* = 4. ^#^*P* < 0.05 vs. control group, *P < 0.05 vs. MPTP group. **(C)** The protein expression level of TH in SNpc of MPTP-treated mice was analyzed by western blot. Data are expressed as means ± SEM. *n* = 4. ^#^*P* < 0.05 vs. control group, *P < 0.05 vs. MPTP group. **(D)** The mRNA expression level of TH in SNpc of MPTP-treated mice was determined by quantitative reverse transcription (qRT)-PCR. Data are expressed as means ± SEM. *n* = 4. ^###^*P* < 0.001 vs. control group, *P < 0.05 vs. MPTP group.

TH is regarded as a marker of dopaminergic neurons (Giráldez-Pérez et al., 2014). In the *in vivo* study, immunohistochemistry analysis of TH expression was applied to investigate the protective effect of Src inhibition on dopaminergic neurons. TH-positive neurons in SNpc showed a significant reduction in MPTP-treated mice, which was prevented by PP2 treatment (Figures 6A,B). We further investigated the expression of TH in SNpc using western blot analysis. Results showed that the mRNA and protein level of TH significantly decreased in SNpc of MPTP-induced PD model mice. Src inhibitor administration markedly increased TH expression (Figures 6C,D), which suggested that the inhibition of Src protected dopaminergic neuron from MPTP-induced neurotoxicity. L-DOPA had no effect on TH expression.

### Src Inhibition Improved the Motor Behavior of the MPTP-Treated Mice

**FIGURE 7:**
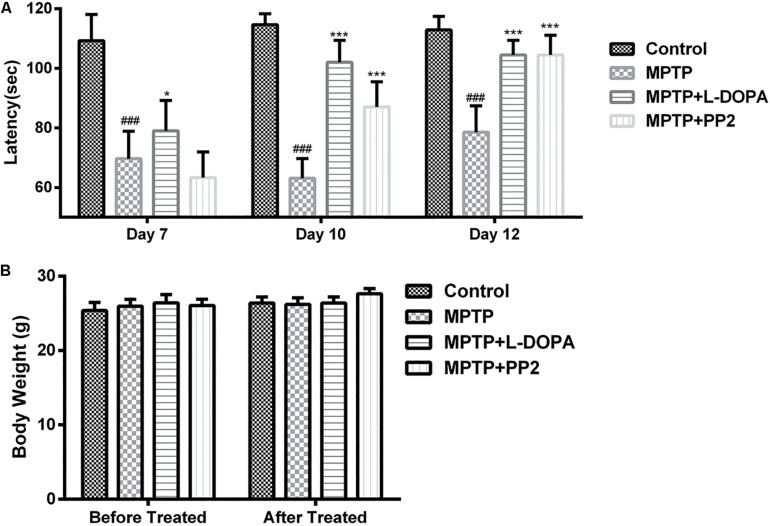
Src inhibition improved the motor behavior of the 1-methyl-4-phenyl-1,2,3,6-tetrahydropyridine (MPTP)-treated mice. Evaluation of mice motor performance using the rotarod test. **(A)** Latency represent time of mice staying on the rod on days 7, 10, and 12. **(B)** Body weight was measured before and after the experiment (at days 1 and 13). Each bar represents the mean ± SEM. ^###^*P* < 0.001 vs. control group, *P < 0.05 and ***P < 0.001 vs. MPTP group.

To determine the effect of Src inhibitor treatment on motor dysfunction, MPTP-induced PD model mice were subjected to rotarod tests at days 7, 10, and 12. Time of mice staying on the rod was used to represent the motor functions of the mice. Our findings revealed that behavioral dysfunction had occurred 7 days post-MPTP injection. Compared with control group, MPTP-treated mice stayed less time on the rod during all the three tests. At day 7, the performance of Src inhibitor treated mice had no improvement, but it enhanced significantly in the tests at days 10 and 12 (Figure 7A). Positive reference L-DOPA improved the behavioral dysfunction of MPTP-treated mice in the all three tests. Body weights measured at the beginning and the end of the dosing period were indistinguishable from control mice or treated mice, which verified that there is no toxicity of PP2 during the experiment (Figure 7B). These data indicated that administration of Src inhibitor had a beneficial effect on the motor dysfunction of MPTP-induced PD model mice.

## Discussion

In this article, we reported the effect of Src inhibition on microglial activation and revealed the role of Src in neuroinflammation. Our results demonstrated that Src inhibition not only decreased the release of inflammatory cytokines but also reduced the loss of dopaminergic neurons. Additionally, Src inhibition could significantly improve the motor behavior of the MPTP-treated mice. Thus, our study suggested that Src might be a potential target for the treatment of PD.

Neuroinflammation that resulted from microglial activation is one of the prominent characteristics of PD (González-Scarano and Baltuch, 1999). Microglial cells are the prime effector cells and resident immune cells of the central nervous system (Nakagawa and Chiba, 2015). Although activated microglia may be beneficial to the host during the early stages in the neurodegenerative process, long-term overactivation of microglia would release various cytokines, chemokines, reactive oxygen species, and reactive nitrogen species (Moss and Bates, 2001). Through all the above substances, uncontrolled overactivation of microglial cells could cause neuronal damage and death (Thameem Dheen et al., 2007); thus, many studies have focused on anti-inflammatory agents because they might help inhibit the progression of this disease. Zhou et al. (2019) found that inhibiting proinflammatory microglia could prevent the progression of Parkinson-like pathology and behavior in a rat model.

During the past decades, Src has received increasing attention in the area of searching targets for inhibiting neuroinflammation. Socodato et al. (2014) found that Src function was essential for triggering microglial cell activation. Furthermore, Yeh et al. (2013) found that modulated Src−ERK1/2−NF-κB pathway could attenuate LPS−induced PGE_2_ and NO production in BV−2 microglial cells. Besides, Hou et al. (2017) demonstrated that Src-Erk-dependent pathway was involved in complement receptor 3-mediated NADPH oxidase activation and dopaminergic neurodegeneration. In addition, our previous study showed that inhibiting Src signaling pathway may have potential anti-neuroinflammatory effects (Wang et al., 2016). All these data revealed that Src played a key role in neuroinflammation.

In the present study, our goal was to verify the relationship between Src and PD-related neuroinflammation and neuropathology. Firstly, we applied the LPS-stimulated BV2 cell model in the *in vitro* experiments. LPS is a bacterial endotoxin that stimulates microglia for the production of NO and many proinflammatory cytokines such as tumor necrosis factor and interleukins (Kirkley et al., 2017). Those proinflammatory cytokines have been widely reported to cause neuroinflammation, lead to neuron death, and deteriorate PD pathology in several animal models (Fellner et al., 2017; Subedi et al., 2017). Src activation is driven in inflammatory cells by proinflammatory cytokines (Yeh et al., 2013). PP2 is a potent inhibitor of Src family tyrosine kinases, and it could inhibit Src phosphorylation in an ATP-competitive manner (Hanke et al., 1996; Roskoski, 2015). Src kinase has two major phosphorylate sites: Tyr416 at the activation loop in the SH1 domain and Tyr527 at the C-terminus. Normally, the basal activity of Src is constitutively inhibited by C-terminal Src kinase (Csk), which phosphorylates Src on Tyr527. Only when Tyr527 is dephosphorylated could Tyr416 auto-phosphorylate and then activate Src by displacing pTyr416 from the binding pocket (Okada, 2012). We found that the phosphorylation level of Src at Tyr416 was increased in LPS-induced BV2 microglia. Apart from microglia, astrocytes, the largest population of glial cells, can also contribute to neuroinflammation and neurodegeneration when converted to a reactive state. We also found that Src expressed in astrocytes and activated following stimulation of astrocytes with LPS and that PP2 inhibited Src activation in activated astrocytes (Supplementary Figure S3). These data confirmed the efficiency of Src inhibitor PP2 in LPS-induced glial activation. Then, the effect of Src inhibition on neuroinflammation was verified. We found that microglial activation and the release of inflammation factors could be decreased by PP2 incubation *via* inhibiting the phosphorylation of Src at Tyr416. Furthermore, conditioned media from the LPS-stimulated BV2 cells could decrease SH-SY5Y cell viability and cause morphological changes, whereas PP2 could significantly attenuate the effects of LPS. Src inhibition in SH-SY5Y could also decrease neurotoxicity induced by conditioned media from the LPS-stimulated BV2, but the underlying mechanism was still unclear. In summary, overactivation of Src in LPS-stimulated condition is bad for neuron survival; thus, Src inhibition could increase cell viability under LPS stimulation.

MPTP-induced *in vivo* PD model was used to evaluate the potential neuroprotection activities and anti-neuroinflammation effects of Src inhibitor. MPTP is a widely used neurotoxin that could primarily kill dopamine-producing neurons in the SNpc (Cleeter et al., 1992). Consistent with other literatures, MPTP did cause neuroinflammation, which is demonstrated by the activation of microglia and the release of inflammatory factors such as IL-6 and TNF-α. Src is an important mediator in several signaling pathways related to neuroinflammation. Therefore, the total protein expression of Src would be increased after MPTP administration. This finding is consistent with other reports (Manocha et al., 2015; Wang et al., 2016). We found that Src inhibition could effectively reduce inflammatory activation of microglia and down-regulate the mRNA levels of inflammatory mediators in the SNpc of MPTP-treated mice. These data indicated that Src tyrosine kinase was involved in MPTP-induced neuroinflammation. Moreover, Src inhibition prevented the reduction of  TH positive neurons and increased the mRNA and protein levels of TH in SNpc of MPTP-treated mice. These evidences supported that inhibiting Src could provide potent neuroprotective effects on dopaminergic neurons. Besides, our study showed that the performances of Src inhibitor-treated mice improved significantly in the behavioral test. Therefore, we suggested that this improvement was related to the anti-inflammatory and neuroprotective effects of Src inhibition. Inflammation involves a variety of signaling pathways, and regulation of Src kinase could not affect all signaling pathway related to inflammation; thus, it does not completely affect all inflammation-related indicators. That is why PP2 showed a partial effect. Owing to the crucial role of inflammation in PD, we thought that the partial effect of PP2 is of great importance. To sum up, these results indicated that the protective effect of dopaminergic neurons and the improvement of motor dysfunction were all attributed to the inhibition of Src and the alleviation of neuroinflammation. Our study provided sufficient evidences to support that Src played an important part in neuroinflammation and neuroprotection.

The development of Src inhibitors for cancer treatment had been carried on for decades (Lowell, 2004). As recent studies found that both tumor cells and tumor-infiltrating immune cells utilize Src to facilitate cancer development and progression (Liu et al., 2014), to some extent, the role that Src inhibitors played in cancer treatment may be attributed to their regulation of inflammation. Hence, clinical data on the use of Src inhibitors in cancer treatment may also provide strong support for their potential application on neuroinflammation-related diseases. Given that Src inhibitors have been developed for cancer treatment, it is normal for the researchers to wonder about the possible risk of side effects in the case of their development for neuroprotection. However, owing to the different doses used, the dose that normally exerts neuroprotection is much lower than the dose used to treat cancer, so it may not be sufficient to cause side effects. Still, these need to be carefully verified in the future. We have checked the effect of PP2 on the control group both *in vitro* and *in vivo* (Supplementary Figure S4). We found that the effect of PP2 is limited in normal cells and animals. Until further evidence is established, clinicians need to be vigilant by ensuring that the use of Src inhibitors remains restricted to their anti-inflammatory effect. To sum up, our study demonstrated that Src kinase inhibition protected dopaminergic neurons against MPTP-induced neurotoxicity owing to its potential anti-neuroinflammatory effects. Therefore, our findings support a new therapeutic strategy that Src family kinases might be a new target for PD treatment.

## Data Availability Statement

All datasets generated for this study are included in the article/Supplementary Material.

## Ethics Statement

The animal study was reviewed and approved by the Animal Care and Welfare Committee of Institute of Materia Medica, Chinese Academy of Medical Sciences and Peking Union Medical College.

## Author Contributions

DZ and HY conceived and designed the experiments, and wrote the manuscript. HY, LW, CZ, YW, JS, ZZ, HL, XB, and XW performed the experiments. HY performed the statistical analysis.

## Conflict of Interest

The authors declare that the research was conducted in the absence of any commercial or financial relationships that could be construed as a potential conflict of interest.

## Abbreviations

CMC-Na: sodium carboxymethyl cellulose
COX2: cyclooxygenase-2
DMEM: Dulbecco’s modified Eagle medium
DMSO: dimethyl sulfoxide
FBS: fetal bovine serum
IBA1: ionized calcium-binding adaptor molecule-1
IL-6: interleukin-6
LPS: lipopolysaccharide
MPTP: 1-methyl-4-phenyl-1,2,3,6-tetrahydropyridine
NO: nitric oxide
PD: Parkinson’s disease
PET: positron emission tomography
SEM: standard error of the mean
SNpc: substantia nigra pars compacta
TH: tyrosine hydroxylase
TNF- α: tumor necrosis factor.
