# Correction: Src inhibition attenuates neuroinflammation and protects dopaminergic neurons in Parkinson's disease models

**DOI:** 10.3389/fnins.2025.1640774

**Published:** 2025-09-29

**Authors:** Hanyu Yang, Lu Wang, Caixia Zang, Yue Wang, Junmei Shang, Zihong Zhang, Hui Liu, Xiuqi Bao, Xiaoliang Wang, Dan Zhang

**Affiliations:** State Key Laboratory of Bioactive Substrate and Function of Natural Medicine, Department of Pharmacology, Institute of Materia Medica, Chinese Academy of Medical Sciences and Peking Union Medical College, Beijing, China

**Keywords:** Src, microglia, neuroinflammation, Parkinson's disease, neuroprotection

In the published article, there were errors in Figure 2A and Figure 5A as published. During assembly of Figure 2A by Adobe Illustrator, a technical error occurred where the DAPI channel in the LPS + PP2-20μM group was partially overlapped by the LPS + PP2-2μM image. However, MERGED panels were not affected. We have provided a corrected version with the proper DAPI alignment.

Due to a mistake during the scanning process of brain slices by the CRO company, one image in Figure 5 from the Model group was inadvertently mislabeled as Control, which caused a mistake in Figure 5A when composing the layouts of the figures. We have re-verified all raw data and replaced the incorrect image with the accurate Control image.

The corrected Figure 2, Figure 5 and their captions appear below.

**Figure 2:**
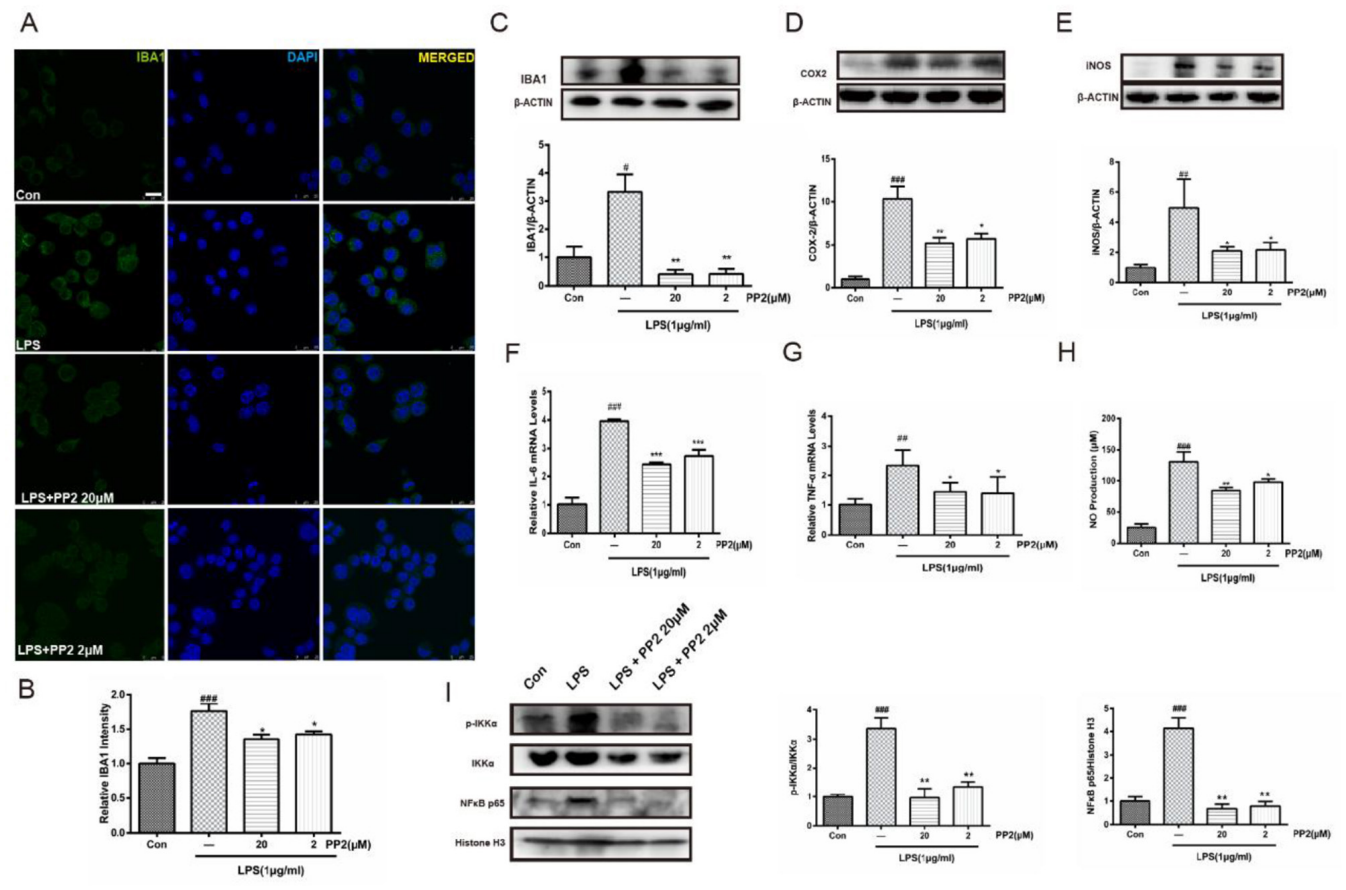
Src inhibition prevented the activation of BV2 microglia and the production of neuroinflammatory molecules subjected to lipopolysaccharide (LPS). **(A)** Cultured BV2 cells were treated with two concentrations of PP2 (2 and 20 μM) in the presence of LPS (1 μg/ml) for 24 h, and then the cells were stained with anti-IBA1 antibody (green) and DAPI stain (scale bar: 8 μm). **(B)** Quantification of the IBA1 staining was provided in a histogram. Each bar represents the mean ± SEM. *n* = 4. ^###^*P* < 0.001 vs. control group, ^*^P < 0.05 vs. LPS group. **(C)** BV2 cells were treated with two concentrations of PP2 (2 and 20 μM) in the presence of LPS (1 μg/ml) for 24 h. The protein level of IBA1 was analyzed by western blot with anti-IBA1 antibody. β-Actin was used as an internal loading control. Each bar represents the mean ± SEM. *n* = 4. ^#^*P* < 0.05 vs. control group, ^**^P < 0.01 vs. LPS group. **(D, E)** The protein level of cyclooxygenase-2 (COX2) and iNOS were examined by western blot. Each bar represents the mean ± SEM. *n* = 4. ^###^*P* < 0.001 and ^##^*P* < 0.01 vs. control group, ^**^P < 0.01 and ^*^P < 0.05 vs. LPS group. **(F, G)** The mRNA levels of IL-6 and TNF-α were analyzed by quantitative reverse transcription (qRT)-PCR. Each bar represents the mean ± SEM. *n* = 4. ^##^*P* < 0.01 and ^###^*P* < 0.001 vs. control group, ^*^P < 0.05 and ^***^P < 0.001 vs. LPS group. **(H)** The level of NO production was determined using the Griess reaction. Each bar represents the mean ± SEM. *n* = 5. ^###^*P* < 0.001 vs. control group, ^*^P < 0.05 and ^**^P < 0.01 vs. LPS group. **(I)** The protein expression level of p-IKKα, IKKα, NF-κB p65, and histone H3 were measured by western blot. Each bar represents the mean ± SEM. *n* = 4. ^###^*P* < 0.001 vs. control group, ^**^P < 0.01 vs. LPS group.

**Figure 5:**
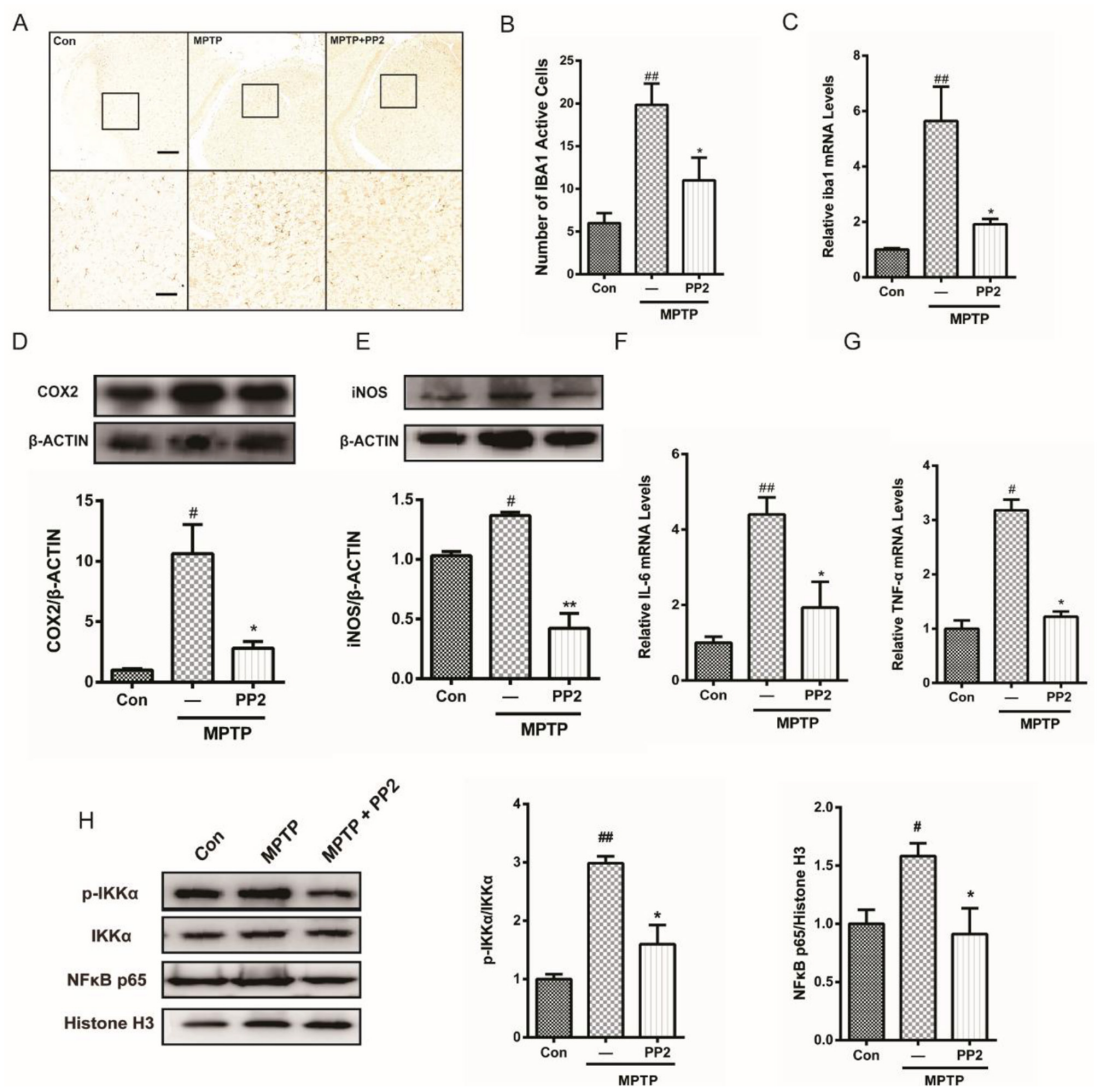
Src inhibition reduced activation of microglial cells and neuroinflammation in 1-methyl-4-phenyl-1,2,3,6-tetrahydropyridine (MPTP)-treated mice. **(A)** The activation of microglia in SNpc showed by IBA1 immunostaining (scale bar: top, 200 μm; bottom, 50 μm). **(B)** The number of IBA1-positive cells per 100 cells was counted and provided in a histogram. Data are expressed as means ± SEM. *n* = 4. ^##^*P* < 0.01 vs. control group^*^P < 0.05 vs. MPTP group. **(C)** The mRNA expression level of IBA1 in SNpc was determined by quantitative reverse transcription (qRT)-PCR. Data are expressed as means ± SEM. *n* = 4. ^##^*P* < 0.01 vs. control group, ^*^P < 0.05 vs. MPTP group. **(D, E)** The protein expression level of cyclooxygenase-2 (COX2) and iNOS were determined by western blot with anti-COX2 and anti-iNOS antibodies. Data are expressed as means ± SEM. *n* = 4. ^#^*P* < 0.05 vs. control group, ^*^P < 0.05 and ^**^P < 0.01 vs. MPTP group. **(F, G)** The mRNA expression level of IL-6 and TNF-α was determined by qRT-PCR. Each bar represents the mean ± SEM. *n* = 4. ^#^*P* < 0.05 and ^##^*P* < 0.01 vs. control group, ^*^P < 0.05 vs. MPTP group. **(H)** The protein expression level of p-IKKα, IKKα, NF-κB p65, and histone H3 were measured by western blot. Each bar represents the mean ± SEM. *n* = 4. ^#^*P* < 0.05 and ^##^*P* < 0.01 vs. control group, ^*^P < 0.05 vs. MPTP group.

In the published article, there were errors in the legends for Figures 4B–D, Figures 5B–E, and 6B, C as published. In these legends, “MPTP group” was mistakenly written as “LPS group”. The corrected legends appear below.

Figure 4. The efficiency of Src inhibitor PP2 was confirmed in 1-methyl-4-phenyl-1,2,3,6-tetrahydropyridine (MPTP)-treated mice. **(A)** The experimental arrangement. **(B–D)** The protein level of p-Src and Src in SNpc of MPTP-treated mice was analyzed by western blot with anti-p-Src and anti-Src antibodies. Data are expressed as means ± SEM. *n* = 4. ^#^*P* < 0.05 vs. control group, ^***^P < 0.001 vs. MPTP group. **(E)** The brown stain represented p-Src-immunoreactive cells in SNpc (scale bar: top, 600 μm; bottom, 25 μm). **(F)** The number of p-Src-positive cells per 100 cells in SNpc was counted and provided in a histogram. Data are expressed as means ± SEM. *n* = 4. ^#^*P* < 0.05 and ^##^*P* < 0.01 vs. control group, ^**^P < 0.01 and ^***^P < 0.001 vs. MPTP group.

Figure 5. Src inhibition reduced activation of microglial cells and neuroinflammation in 1-methyl-4-phenyl-1,2,3,6-tetrahydropyridine (MPTP)-treated mice. **(A)** The activation of microglia in SNpc showed by IBA1 immunostaining (scale bar: top, 200 μm; bottom, 50 μm). **(B)** The number of IBA1-positive cells per 100 cells was counted and provided in a histogram. Data are expressed as means ± SEM. *n* = 4. ^##^*P* < 0.01 vs. control group, ^*^P < 0.05 vs. MPTP group. **(C)** The mRNA expression level of IBA1 in SNpc was determined by quantitative reverse transcription (qRT)-PCR. Data are expressed as means ± SEM. *n* = 4. ^##^*P* < 0.01 vs. control group, ^*^P < 0.05 vs. MPTP group. **(D, E)** The protein expression level of cyclooxygenase-2 (COX2) and iNOS were determined by western blot with anti-COX2 and anti-iNOS antibodies. Data are expressed as means ± SEM. *n* = 4. ^#^*P* < 0.05 vs. control group, ^*^P < 0.05 and ^**^P < 0.01 vs. MPTP group. **(F, G)** The mRNA expression level of IL-6 and TNF-α was determined by qRT-PCR. Each bar represents the mean ± SEM. *n* = 4. ^#^*P* < 0.05 and ^##^*P* < 0.01 vs. control group, ^*^P < 0.05 vs. MPTP group. **(H)** The protein expression level of p-IKKα, IKKα, NF-κB p65, and histone H3 were measured by western blot. Each bar represents the mean ± SEM. *n* = 4. ^#^*P* < 0.05 and ^##^*P* < 0.01 vs. control group, ^*^P < 0.05 vs. MPTP group.

Figure 6. Src inhibition enhanced the survival of dopaminergic neurons of the 1-methyl-4-phenyl-1,2,3,6-tetrahydropyridine (MPTP)-treated mice. **(A)** Representative images showed tyrosine hydroxylase (TH)-immunoreactive neurons in the SNpc (scale bar: top, 500 μm; bottom, 250 μm). **(B)** The number of TH-positive neurons per slide in SNpc was counted for each section and provided in a histogram. Data are expressed as means ± SEM. *n* = 4. ^#^*P* < 0.05 vs. control group, ^*^P < 0.05 vs. MPTP group. **(C)** The protein expression level of TH in SNpc of MPTP-treated mice was analyzed by western blot. Data are expressed as means ± SEM. *n* = 4. ^#^*P* < 0.05 vs. control group, ^*^P < 0.05 vs. MPTP group. **(D)** The mRNA expression level of TH in SNpc of MPTP-treated mice was determined by quantitative reverse transcription (qRT)-PCR. Data are expressed as means ± SEM. *n* = 4. ^###^*P* < 0.001 vs. control group, ^*^P < 0.05 vs. MPTP group.

The original version of this article has been updated.

