# Supplementary material for: Src Inhibition Attenuates Neuroinflammation and Protects Dopaminergic Neurons in Parkinson’s Disease Models
Source: Front Neurosci. 2020 Feb 18;14:45. doi: 10.3389/fnins.2020.00045 (PMC7040487; doi:10.3389/fnins.2020.00045)
Supplement: Supplementary file 1 [file Presentation_1.PDF]

Supplementary materials

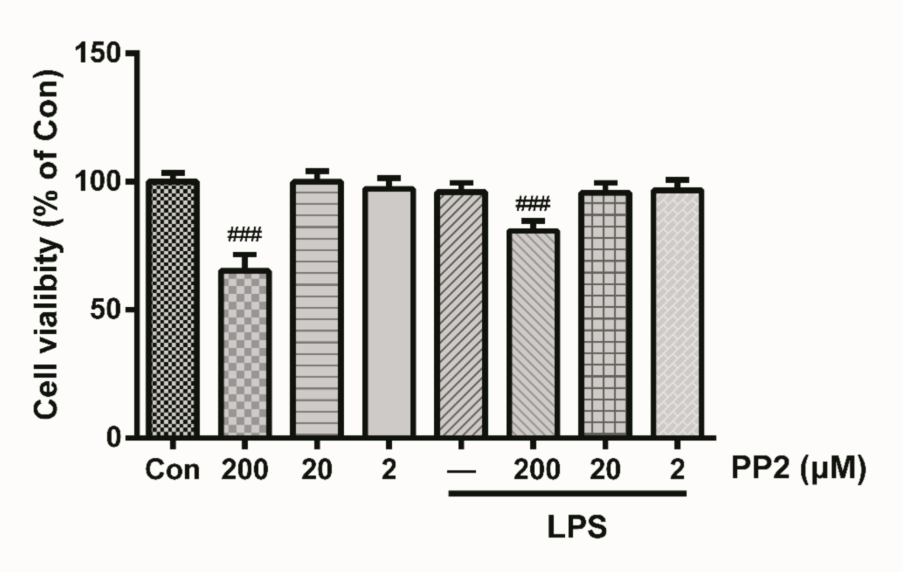

Figure S1

Cultured BV-2 microglia were treated with three concentrations of PP2 (2 to 200 μM) in the presence or absence of LPS (1 μg/ml) for 24 h. MTT assay was used to measure cell viability. Data are expressed as means ± SEM. n=4. ###P < 0.001 vs. Control group

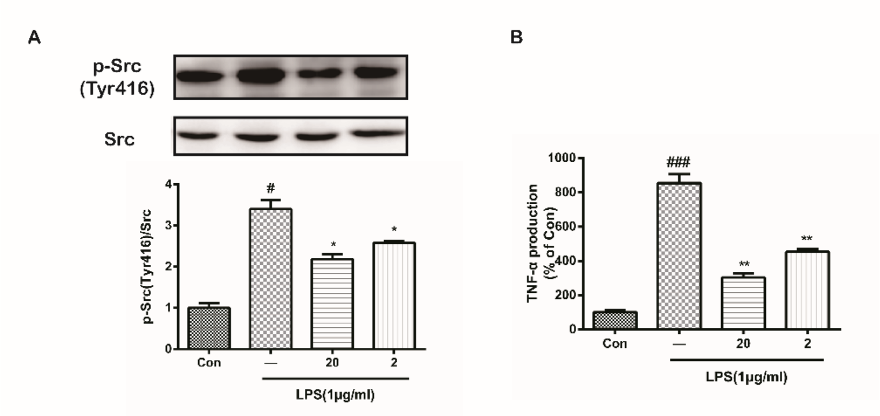

Figure S2

Primary microglial cells were treated with PP2 (2 and 20 μM) in the presence or absence of LPS (1 μg/ml) for 6 h. Western blot was used to measure the protein expression. Data are expressed as means ± SEM. n=3. #P < 0.5 vs. Control group, *P < 0.05 vs. LPS group. ELISA essay was used to measure the concentration of TNF-α. ###P < 0.05 vs. Control group, **P < 0.01 vs. LPS group

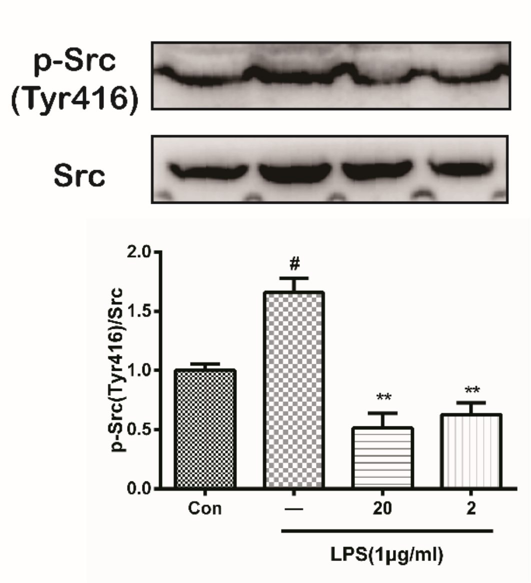

Figure S3

Cultured C6 astrocytes were treated with PP2 (2 and 20 μM) in the presence or absence of LPS (1 μg/ml) for 24 h. Western blot was used to measure the protein expression. Data are expressed as means ± SEM. n=4. #P < 0.05 vs. Control group, **P < 0.01 vs. LPS group

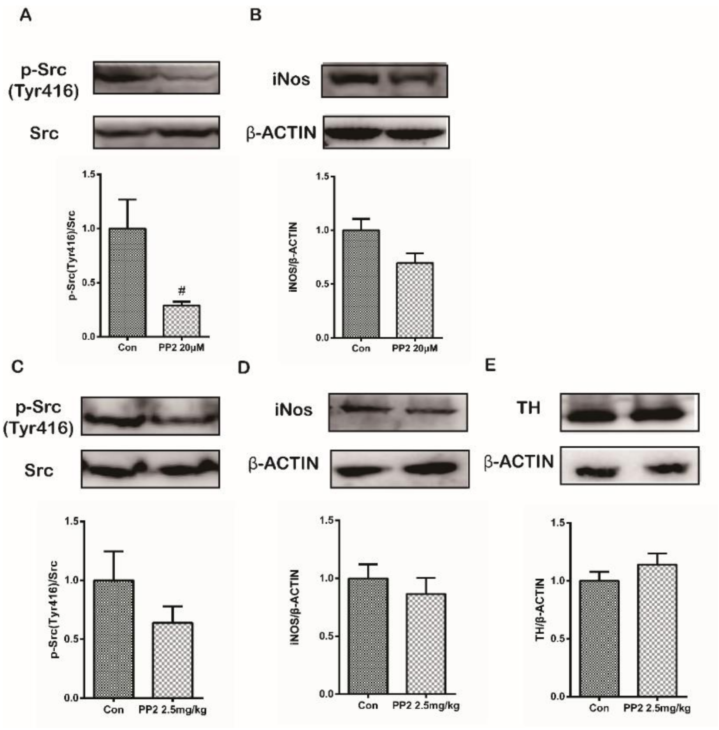

FigureS4

(A,B) Cultured BV-2 microglia were treated with PP2 (20 μM) for 24 h. The protein level of p-Src, Src, iNos and β-Actin was analyzed by weatern blot. **(C,D,E)** The protein level of p-Src, Src,iNos, β-Actin and TH of MPTP-treated mice was analyzed by western blot with anti-p-Src and anti-Src antibodies. Data are expressed as means ± SEM. n=4. #*P* < 0.05 vs. Control group,
